# A Single LiDAR-Based Feature Fusion Indoor Localization Algorithm

**DOI:** 10.3390/s18041294

**Published:** 2018-04-23

**Authors:** Yun-Ting Wang, Chao-Chung Peng, Ankit A. Ravankar, Abhijeet Ravankar

**Affiliations:** 1 Department of Aeronautics and Astronautics, National Cheng Kung University, Tainan 701, Taiwan; omiwanggg@gmail.com; 2 Division of Human Mechanical Systems and Design, Faculty of Engineering, Hokkaido University, Sapporo 060-8628, Japan; ankit@eng.hokudai.ac.jp; 3 Lab of Smart Systems Engineering, Kitami Institute of Technology, Hokkaido, Kitami 090-8507, Japan; abhijeetravankar@gmail.com

**Keywords:** indoor localization, pose estimation, iterative closet point, SLAM, LiDAR

## Abstract

In past years, there has been significant progress in the field of indoor robot localization. To precisely recover the position, the robots usually relies on multiple on-board sensors. Nevertheless, this affects the overall system cost and increases computation. In this research work, we considered a light detection and ranging (LiDAR) device as the only sensor for detecting surroundings and propose an efficient indoor localization algorithm. To attenuate the computation effort and preserve localization robustness, a weighted parallel iterative closed point (WP-ICP) with interpolation is presented. As compared to the traditional ICP, the point cloud is first processed to extract corners and line features before applying point registration. Later, points labeled as corners are only matched with the corner candidates. Similarly, points labeled as lines are only matched with the lines candidates. Moreover, their ICP confidence levels are also fused in the algorithm, which make the pose estimation less sensitive to environment uncertainties. The proposed WP-ICP architecture reduces the probability of mismatch and thereby reduces the ICP iterations. Finally, based on given well-constructed indoor layouts, experiment comparisons are carried out under both clean and perturbed environments. It is shown that the proposed method is effective in significantly reducing computation effort and is simultaneously able to preserve localization precision.

## 1. Introduction

Simultaneous localization and mapping (SLAM) is a method of building a map under exploration and estimating vehicle pose based on sensor information in an unknown environment. When exploring an unpredictable environment, an unmanned vehicle is generally employed for exploration as well as localization. In this regard, the vehicle could be equipped with a single sensor for detecting and identifying surroundings, or by attaching two or even more sensors on the vehicle to enhance its estimation capability.

When considering different kinds of sensors [1], laser range finders (LRFs), vision, and Wi-Fi networks are popular sensing techniques for indoor localization tasks. Recently, with advancement in computer vision and image processing, many researchers have started investigating the vision-based SLAM [2,3]. Under the condition that the captured images are matched sufficiently, features can be extracted using Scale-Invariant Feature Transform (SIFT) [4] or Speeded Up Robust Features (SURF) [5]. Other indoor localization methods consider the amplitude of received signal from Wi-Fi networks [6,7,8,9,10]. These localization strategies depend on pre-installed wireless hardware devices on the site and thus may not be applicable in Wi-Fi denied environments.

For an ultra-low-cost SLAM module, previous works have considered a set of on-board ultrasonic sensors [11], which provided sparse measurements about the environment. However, the robot pose might lose its pose under complicated environments without the aid of robot kinematics information. To achieve robust image recognition [12], robot navigation and map construction, the depth sensor, Kinect v2, was considered [13]. Kinect v2 is based on the time-of-flight measurement principle and can be used in outdoors environment. Since the multi-depth sensors are able to provide highly dense 3D data [14], the real-time computation effort is relative higher. Furthermore, for well-constructed indoor environment, such hardware configuration is not necessary for 2D robot positioning.

Owing to the light weight and portable advantage, light detection and ranging (LiDAR) has attracted more and more attention [15,16]. LiDAR possesses a high sampling rate, high angular resolution, good range detection, and high robustness against environment variability. As a result, in this research, a single LiDAR is used for indoor localization.

By analyzing the position of features in each frame at every movement of the vehicle, one can figure out the vehicle’s traveling distance and heading. With different scanning data, an iterative closed point (ICP) [17] algorithm is employed to find the most appropriate robot pose matching conditions, including rotation and translation. However, the ICP may not always lead to good pattern matching if point cloud registration issue is not well addressed. In other words, a better point registration will lead to better robot pose estimation. To address this, point cloud outliers must be identified and recognized. Another issue when applying the ICP is computation efficiency. Since the ICP algorithm considers the closest-point rule to establish correspondences between points in current scan and a given layout, the searching effort can increase dramatically when the scan or a layout contains large amounts of data.

The researches [18,19,20,21,22,23,24] has addressed and solved some of the problems when applying ICP, including (1) wrong point matching for large initial errors, (2) expensive correspondence searching, (3) slow convergence speed, and (4) outlier removal. For robot pose subjected to large initial angular displacement, especially in [16], iterative dual correspondence (IDC) is proposed. However, it demands higher computation due to its dual-correspondence process. Metric-based ICP (MbICP) [21] considers geometric distance that takes translation and rotation into account simultaneously. The correspondence between scans is established with this measure and the minimization of the error is carried out in terms of this distance. The MbICP shows superior robustness in the case of existing large angular displacement. Among various planar scan matching strategies, Normal Distribution Transformation (NDT) [22] and Point-to-Line ICP (PLICP) [23] illustrate state-of-the-art performance in consideration of scan matching accuracy. NDT transforms scans onto a grid space and tends to find the best fit by maximizing the normal distribution in each cell. The NDT does not require point-to-point registrations, so it enhances matching speed. However, the selection of the grid size dominates estimation stability. The PLICP considers normal information from environment’s geometric surface and tries to minimize the distance projected onto the normal vector of the surface. In addition, a close-form solution is also given such that the convergence speed can be drastically improved.

To obtain precise and high-bandwidth robot pose estimation, the vehicle’s dynamics and traveling status can be further integrated into a Kalman filter [25]. A Kalman filter provides the best estimate to eliminate noise and provides a better robot pose prediction. Other researchers have considered the wheel odometry fusion-based SLAM [26,27], which integrates robot kinematics and encoder data for pose estimation. However, it might not be suitable for realization of a portable localization system.

Based on the aforementioned issues, in this work, the LiDAR is considered as the only sensor for mapping and localization. To avoid mismatched point registration and to enhance matching speed, we propose a feature-based weighted parallel iterative closed point (WP-ICP) architecture inspired by [18,23,28,29,30,31]. The main advantages of the proposed method are as follows: (a) Point sizes of the model set and data set are significantly reduced so that the ICP speed can be enhanced. (b) A split and merge algorithm [28,29] is considered to divide the point cloud into two feature groups, namely, corner and line segments. The algorithm works by matching points labeled as corners to the corner candidates; similarly, for those points labeled as lines can only be matched to the lines candidates. As a result, it attenuates any possibilities of point cloud mismatch.

In this paper, it is supposed that the well-constructed indoor layout is given in advance. The main design object is to reduce the computation effort and to maintain the indoor positioning precision. The rest of this paper is organized as follows. In Section 2, an adaptive breakpoint detector is firstly introduced for scan point segmentation. A clustering algorithm and a split–merge approach is further considered for point clustering and feature extraction, respectively. In Section 3, a WP-ICP algorithm is proposed. Section 4 presents real experiments to evaluate the effectiveness of the proposed method. Finally, Section 5 outlines conclusions and future work.

## 2. Feature Extraction

For feature extraction, a robot is employed in an indoor environment and moves in a given layout, and a localization algorithm is introduced. In addition, real-time sensing and pose estimation is key for practical realization. Feature extraction plays an important role in reducing the amount of point cloud data for computational speedup.

### 2.1. The Main Concept of Feature Extraction

Due to the high scan resolution of LiDAR and the given layout, construction of a KD-Tree will be highly time-consuming if all data points are fed into the ICP algorithm. Therefore, extracting informative feature points to represent the environment is one of the important tasks.

**Figure 1:**
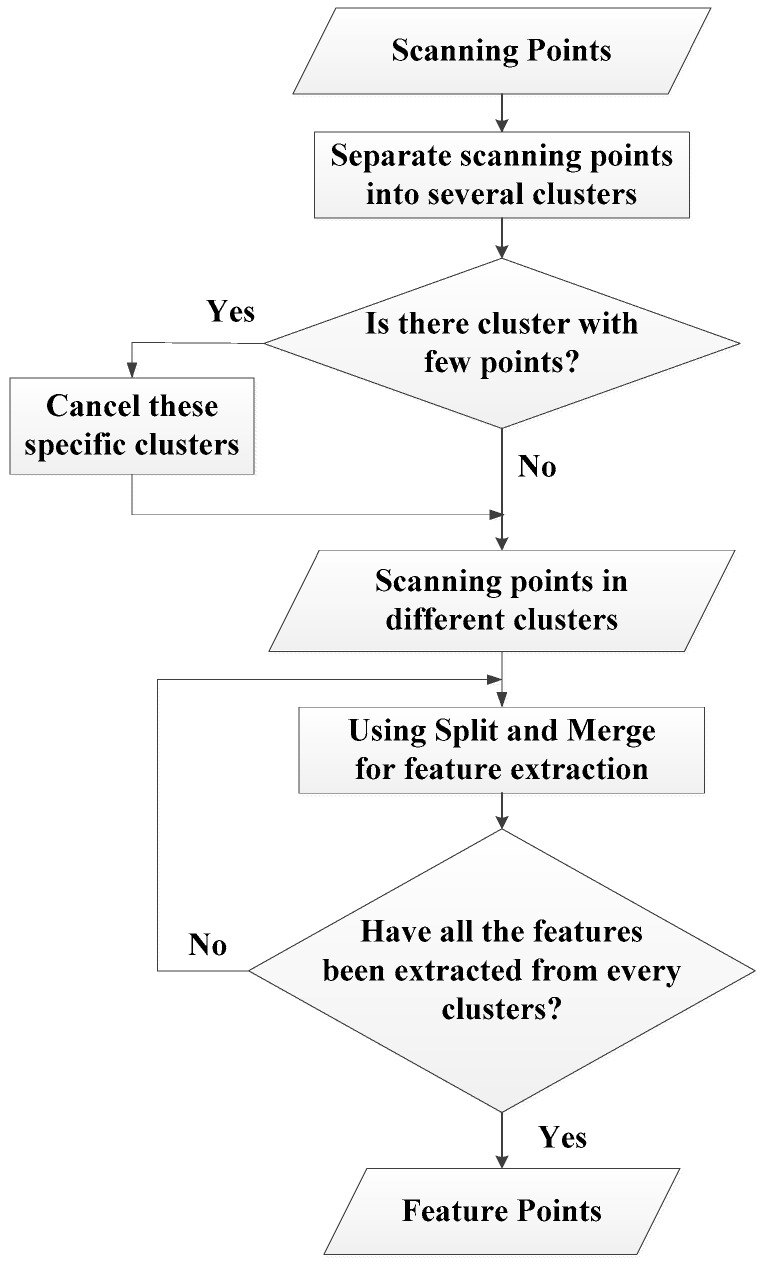
Flow chart of feature extraction.

In this research, a feature extraction scan matching procedure is proposed as summarized in Figure 1. Firstly, all the scanning points are separated into many clusters by invoking the adaptive breakpoint detector (ABD) [28]. The main idea of an ABD is to find the breakpoints in each scan, where the scanning direction of a LiDAR is counterclockwise and continuous. Therefore, by detecting breakpoints, the algorithm can determine if there exists a discontinuity between two consecutive scanning points. However, the threshold for the breakpoint determination should be adaptive with respect to the scanning distance, which is presented in the following subsection.

### 2.2. A Novel Method for Finding Clusters

Consider that, for each scan, there exists n beams. The radius for each beam is denoted as $r_{i}$, where i=1,⋯,n. To enhance the clustering performance, a simple and straightforward adaptive radius clustering (ARC) algorithm is developed as follows:

(1) $$\Delta S=R\times \Delta \theta =R\times (\alpha \times 2\pi /360),\;R=\min\left(r_{i},r_{i-1}\right)$$

(2) λ=N×ΔS

where ΔS denotes an adaptive arc length between points, α is the angular resolution provided by LiDAR specification, N is a design scaling factor relative to LiDAR noise level, and λ is the threshold for clustering. Therefore, if the distance between two scanning points in the same scan is larger than λ, those points are going to be divided into two different clusters; that is

(3) $$\left\|p_{i}-p_{i-1}\right\|>\lambda$$

**Figure 2:**
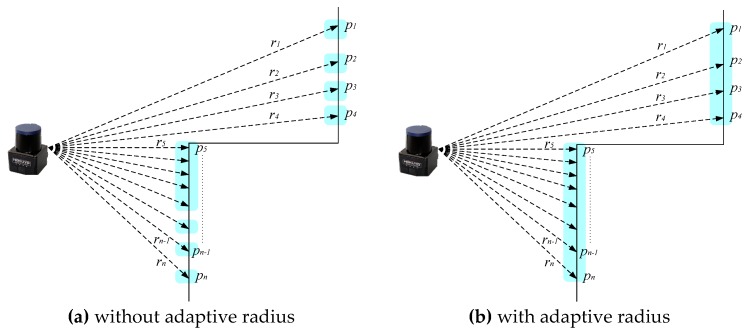
Illustration of the adaptive radius clustering (ARC) algorithm.

Based on the ARC, the scan shown in Figure 2b can be clustered into two groups. Else, it may be divided into several segments due to the radius variations as illustrated in Figure 2a. Therefore, the ARC algorithm is able to separate clusters according to LiDAR’s measurement characteristics.

After utilizing the ARC algorithm, clusters with fewer scanning points are treated as outliers. For clusters with lower density points that cannot provide reliable information are discarded before the feature extraction step.

### 2.3. Split and Merge for Corner and Line Extractions

Comparing a few of the feature extraction methods in different aspects, including the speed of the algorithm, correctness, and so on, the split and merge [15,28,29] is considered for feature extraction in this research. The algorithm is capable of extracting corner feature points in the environment, which can be then taken as stable and reliable feature points.

**Figure 3:**
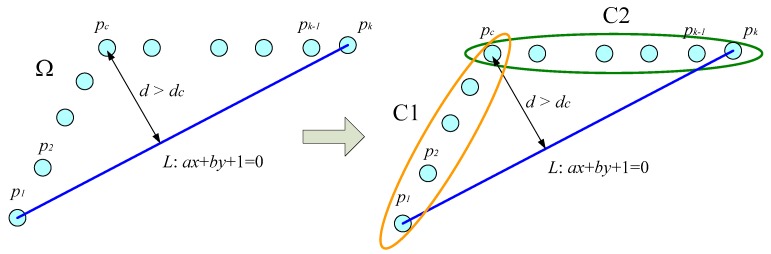
Illustration of the split and merge scheme.

The splitting procedure also combines the idea of Iterative End Point Fitting (IEPF) [30,32], which is a recursive algorithm for extracting features. For a cluster $\Omega =\left\{x_{i},y_{i}|i=1,\cdots ,k\right\}$, the split and merge algorithm connects the first point and the last point to form a straight line. The algorithm then calculates deviations from all the points to this line. If a point where the corresponding maximum distance is larger than a predefined deviation threshold $d_{c}$, this point is labeled as a feature point and it further splits the cluster into two subsets. The feature point $P_{c}\left(x_{i},y_{i}\right)\in \Omega$ can be determined by the following rule:

(4) $$P_{c}\left(x_{i},y_{i}\right)\leftarrow d:=\underset{P_{c}\in \Omega }{\arg\max}\frac{\left|ax_{i}+by_{i}+1\right|}{\sqrt{a^{2}+b^{2}}}>d_{c}$$

where a,b are the coefficients of a line equation L:ax+by+1=0, which is composed of $P_{1}$ and $P_{k}$. Figure 3 demonstrates the main process of the split and merge algorithm. The algorithm recursively splits the set of points into two subsets until the condition, Equation (4), is not satisfied.

## 3. The Proposed Method: WP-ICP

### 3.1. Pose Estimation Algorithm

SLAM is considered to be a chicken and egg problem since precise localization needs a reference map, and a good mapping result comes from a correct estimation of the robot pose [33]. To achieve SLAM, these two issues must be solved simultaneously [34]. However, in this work, only localization is considered. Therefore, by assuming that a complete layout of the environment is given in advance, a novel scan matching algorithm is presented and will be introduced in the next subsection.

Suppose that the correspondences are already known, the pose estimation can be considered as an estimate of rigid body transform, which can be solved efficiently via singular value decomposition (SVD) technique [35].

Let $P=\left\{p_{1},p_{2},\cdots p_{N}\right\}$ be data set from a current scan and $Q=\left\{q_{1},q_{2},\cdots q_{N}\right\}$ be a model set received from a given layout. The goal is to find a rigid body transformation pair (R,t) such that the best alignment can be achieved in the least error sense. It can be stated as

(5) $$\left(R,t\right)=\arg\min\sum_{i=1}^{N}w_{i}{\left\|Rp_{i}+t-q_{i}\right\|}^{2}$$

where $w_{i}$ are the weights for each point pair.

The optimal translation vector can be calculated by

(6) $$t=\bar{q}-R\bar{p}$$

where

(7) $$\bar{p}=\frac{\sum_{i=1}^{N}w_{i}p_{i}}{\sum_{i=1}^{N}w_{i}},\bar{q}=\frac{\sum_{i=1}^{N}w_{i}q_{i}}{\sum_{i=1}^{N}w_{i}}$$

can be taken as weighted centroids for the data set and the model set, respectively.

Let $x_{i}=p_{i}-\bar{p}$ and $y_{i}=q_{i}-\bar{q}$. Consider also matrices X, Y, and W which are defined by $X=\left[\begin{array}{ccc} x_{1} & \cdots & x_{N} \end{array}\right]$, $Y=\left[\begin{array}{ccc} y_{1} & \cdots & y_{N} \end{array}\right]$, and $W=diag\left(w_{1},\cdots ,w_{N}\right)$, respectively.

Defining $S={\mathrm{XWY}}^{T}$ and then applying the singular value decomposition (SVD) on S yields

(8) $$S=U\;\Sigma \;V^{T}$$

where U and V are unitary matrices, and Σ is a diagonal matrix. It has been proved that the optimal rotation matrix is available by considering

(9) $$R={\mathrm{VU}}^{T}$$

Based on Equation (9), the translational vector given in Equation (6) can also be solved.

**Figure 4:**
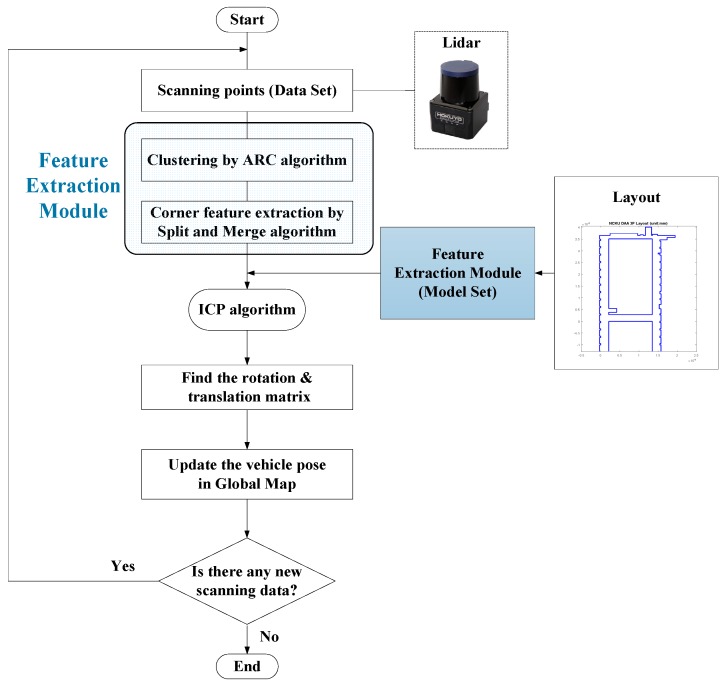
Corner-feature-based pose estimation.

According to the ARC, the split–merge algorithm, and the ICP, the procedure of the corner-feature-based ICP pose estimation is summarized in Figure 4. To reject the outlier during the feature point registration, the weightings $w_{i}$ can be designed according to the Euclidean distance, and the values for certain unreasonable feature pairs can be set to zero.

### 3.2. A Weighted Parallel ICP Pose Estimation Algorithm

An environment that has a similar layout generally results in good estimates. However, in practice, it is not always the case. To verify the feasibility of the proposed method, the proposed algorithm needs to be robust enough even in the presence of environment uncertainties such as moving people.

Based on the results presented in Section 2 and Section 3, a WP-ICP is proposed. The WP-ICP considers two features for point clouds that are pre-processed: one is the corner feature and the other is the line feature. Corners are important feature points in the environment as they are distinct, whereas walls are stable feature points and a good candidate for feature extraction in structured environments [15,31,36]. Taking advantage of LiDAR for detecting surroundings, walls can be represented as line segments, which are composed of two corners. Furthermore, we also considered the center point of a line segment as another matching reference point.

**Figure 5:**
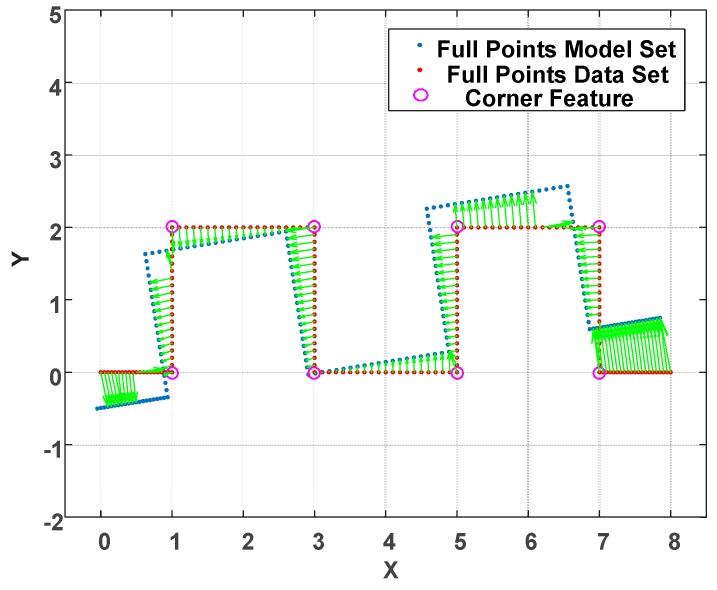
Incorrect point registration caused by the nearest neighbor search (NNS) Iterative Closest Point (ICP) algorithm.

The motivation for such features are that indoor environments, e.g., offices and buildings, are generally well structured. Therefore, feature-based localization is suitable for such environments. Examining the traditional full-points ICP algorithm, point cloud registration is achieved by means of the nearest neighbor search (NNS) concept. There is no further information attached to those points. Therefore, it is easy to obtain incorrect correspondence as shown in Figure 5. Under this circumstance, several iterations are usually needed to converge the point registration. In addition, the ICP gives rise to an obvious time cost for registration especially when the size of point cloud is large. To solve the incorrect correspondence and iteration time cost issues, a feature-based point cloud reduction method is developed.

**Figure 6:**
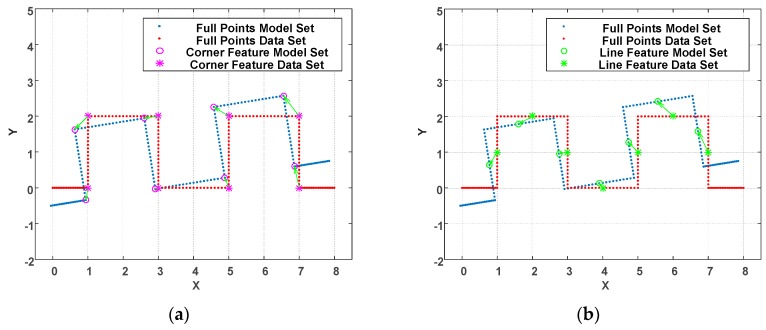
Illustration of feature-based point cloud registration. (**a**) Corner features are matched to corresponding corner features (**b**) Line features are matched to corresponding line features.

For the proposed WP-ICP, the point cloud is first characterized by fewer corners and lines. This has two main advantages: (a) First, the size of the point cloud is reduced significantly and thus enhances the ICP speed. (b) Second, the polished points are labeled as corner or line features. Only those points labeled as corners can be matched to the corner candidates; similarly, only those points labeled as lines can be matched to the lines candidates. The result is shown in Figure 6. Figure 6a illustrates the matching condition for corners while Figure 6b represents the matching condition for lines. The parallel-ICP results in correct point registration and thereby reduces the number of iterations.

The main advantage of the WP-ICP is that the number of data points in a data set as well as a model set can be significantly reduced. On the contrary, the full-points ICP algorithm includes all data points for correspondence searching and thus leads to low computation efficiency.

Moreover, in the proposed WP-ICP, the scan points are clustered into the corner or the line groups, respectively. Based on the parallel mechanism, the points from a corner set can never be matched with the points from a line set. It can thus avoid mismatching during the point registration. However, for full point ICP, many mismatches could happen once the distances between those two set points are close enough.

Since the WP-ICP has two ICP processes at the same time, it generates two pairs of robot pose, namely $\left(R_{C},t_{C}\right)$ and $\left(R_{L},t_{L}\right)$. Therefore, it is desired to fuse these two poses to come out with a more confident pose estimate.

The criterion for the confidence evaluation is designed as follows:

(10) $$\Gamma_{C}=\frac{\#\mathrm{of}\mathrm{matched}corner\mathrm{feature}\mathrm{points}}{\#\mathrm{of}\mathrm{total}corner\mathrm{feature}\mathrm{points}},\Gamma_{L}=\frac{\#\mathrm{of}\mathrm{matched}line\mathrm{feature}\mathrm{points}}{\#\mathrm{of}\mathrm{total}line\mathrm{feature}\mathrm{points}}$$

where $\Gamma_{C,L}$ represent the confidences and can be treated as fused weights for corner feature ICP and line feature ICP, respectively.

The final step is to calculate a fused pose estimate $\left(R_{fused},t_{fused}\right)$ as follows:

(11) $$\begin{array}{l} R_{fused}=\alpha R_{C}+\left(1-\alpha \right)R_{L}\\ t_{fused}=\alpha t_{C}+\left(1-\alpha \right)t_{L} \end{array},\;\alpha =\frac{\Gamma_{C}}{\Gamma_{C}+\Gamma_{L}}$$

where the heading rotation angle is used to obtain the $R_{fused}$.

**Figure 7:**
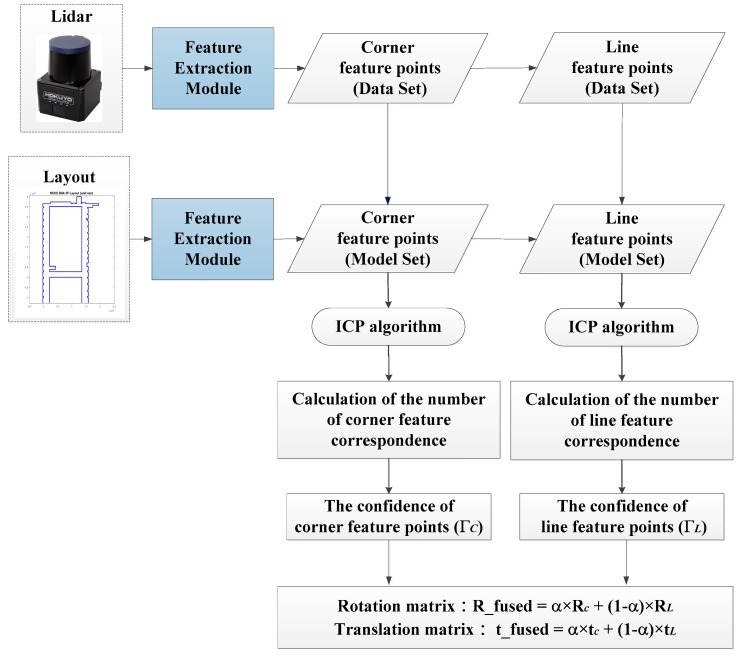
The weighted parallel iterative closed point (WP-ICP) pose estimation algorithm.

Since the WP-ICP provides two sources of feature points, the real-time LiDAR scanning points are going to be separated into two groups including corners and lines. Each group is then matched with its corresponding features. Based on this parallel matching mechanism, serious mismatching can be avoided, resulting in improved localization stability and precision. The flow chart of the WP-ICP is illustrated in Figure 7.

The practical benefits of the proposed WP-ICP include the following: (a) computation effort is reduced when KD-Trees are built and nearest point registration is applied; (b) correct point cloud registration significantly reduces ICP iterations, enabling fast robot pose estimate; (c) robot pose is determined by feature-based ICP fusion of two features (corner and line), which makes the estimate less sensitive to uncertain environments.

## 4. Experiments and Discussions

**Figure 8:**
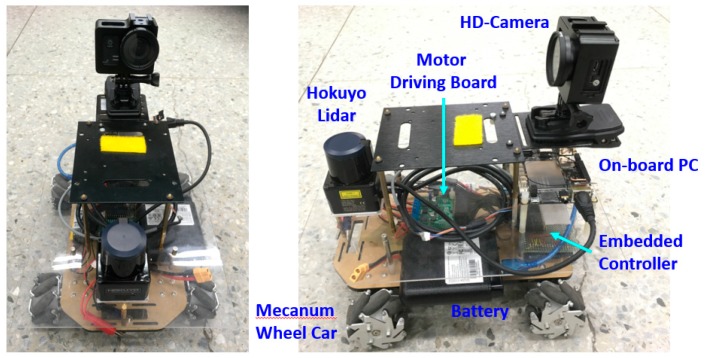
A LiDAR-based portable module and a moving platform.

For the experiments, a Hokuyo UST-20LX Scanning Laser Rangefinder is used, where a 20 Hz scan rate and a 15 m scan distance are applied. The scanning angle resolution is 0.25°. Based on these settings, the maximum scanning points are 1081 point per scan; that is, 20 × 1081 = 21,620 points per second. The experiment system is shown in Figure 8, which includes (1) a portable LiDAR module (the upper half part) and (2) a mecanum wheeled robot (the lower half part). In this work, since the LiDAR is considered as the single sensor, the robot vehicle is only taken as a moving platform. There is no communication between the LiDAR module and the vehicle. The maximum moving speed of the vehicle in the following experiments is restricted to 50 cm/s.

Based on the resolution and testing result, *N* in Equation (2) is set to 15. With regard to the ARC, clusters containing fewer than 5 points are considered as outliers and are removed before the WP-ICP is applied. The distance *d_c_* = 10 cm is used for the split and merge process. For each iteration, the weights for ICP will be set to zero if the distances between the points’ correspondences are greater than 50 cm. This threshold is determined in accordance with the maximum moving speed of the robot.

**Figure 9:**
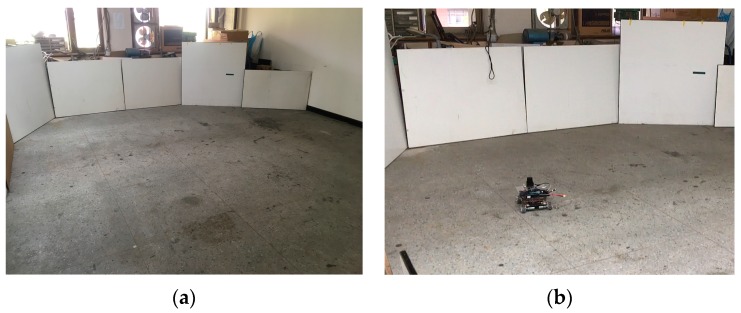

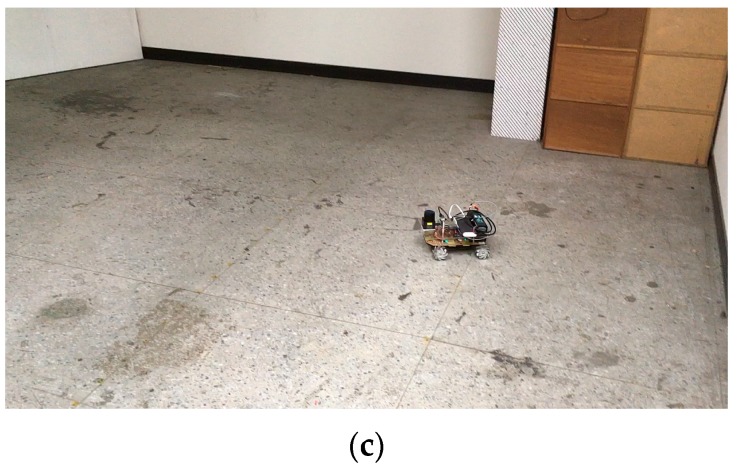
Scene-1: a robot moves in a clear environment with no obstacles. (**a**) Experimental environment. (**b**) Robot is moved in a guided rectangular path. (**c**) Robot is moved randomly.

To ensure the WP-ICP algorithm is feasible, an experiment was firstly carried out in a clear environment with no obstacles, which is shown in Figure 9. The area of the testing environment was about 5 m × 6 m. There are two experiments that were carried out in this environment. One is guiding the vehicle in a rectangular path and the other is moving the vehicle randomly.

**Figure 10:**
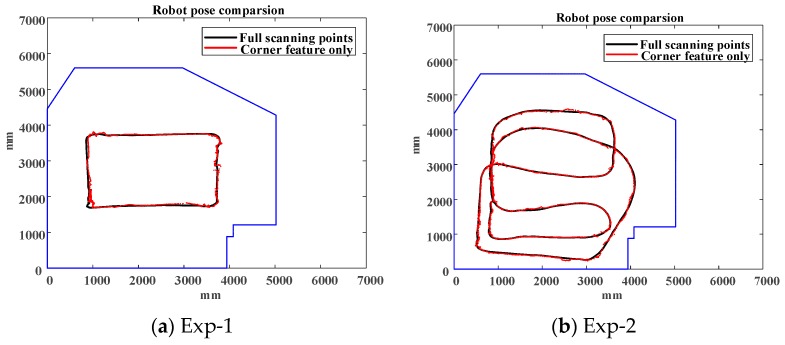
Different algorithms for the first/second experiments.

**Figure 11:**
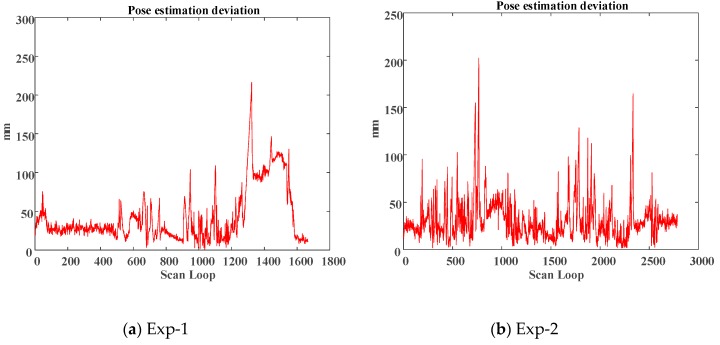
Pose estimation deviation comparison of the first/second experiments.

Considering full points based ICP (shown in the black line) result as the ground truth, using corner feature only (shown in the red line) is able to result in an accurate pose estimate in a clear environment as illustrated in Figure 10. Figure 11 shows the deviation comparisons of the pose estimation and the average of deviations are 3.06786 and 4.29678 cm, respectively.

**Figure 12:**
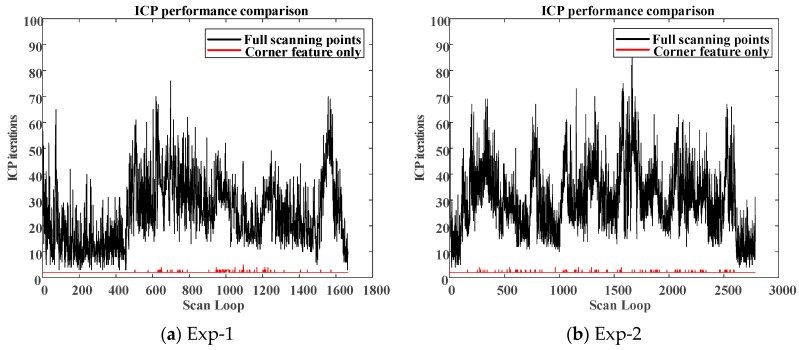
ICP performances of the first/second experiments.

Moreover, to compare the real-time estimation capabilities between the full-points ICP and the corner-feature-based ICP, the total number of ICP iteration at each LiDAR scan loop is addressed. Less ICP iterations are helpful for real-time realization. Figure 12 shows the number of ICP iterations between different algorithms for different experiments. It should be noted that, when using corner as the only feature, the maximum iteration loop in the ICP was no more than 4 (two iterations on average). However, using full-points ICP results in more than 25 iterations on average. In this environment, the WP-ICP was also applied. The localization performance is very close to the one conducted by the corner-based ICP. Therefore, the main advantage of the WP-ICP is not obvious under this clear environment.

**Figure 13:**
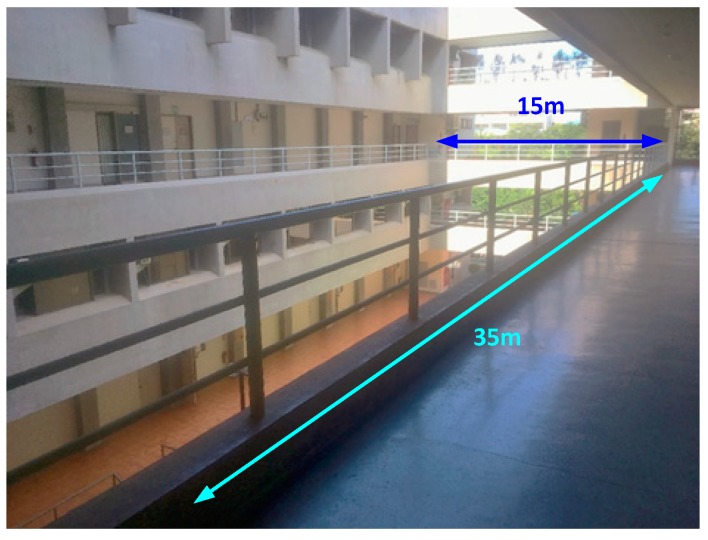
Scene-2: the 3rd floor, Department of Aeronautics and Astronautics, NCKU.

**Figure 14:**
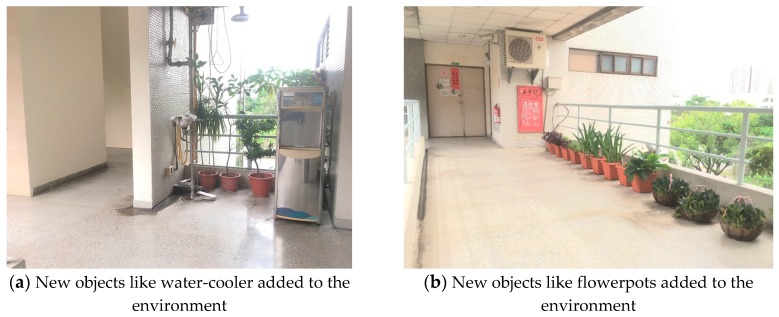
DAA-3F local snapshot with new objects to test the robustness of the algorithm.

To illustrate the superior pose estimation robustness against the corner-based ICP, another experiment has been carried out on the 3rd floor of Department of Aeronautics and Astronautics, NCKU, shown in Figure 13. The area is about 15 × 40 m^2^ and contained different sized objects like flowerpots and water-cooler as shown in Figure 14, which can be taken as unknown disturbances for post estimation. These objects were added later in the environment, and hence can be used to test the feasibility and robustness of the proposed WP-ICP algorithm in dynamic environments.

**Figure 15:**
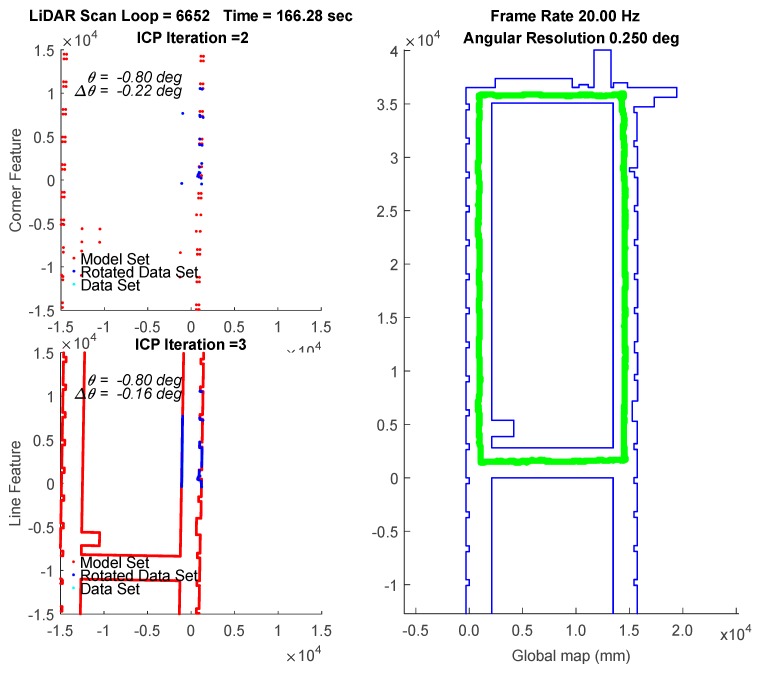
Localization by the WP-ICP with 10 cm interpolation. Upper left corner subplot: corner features. Lower left corner subplot: line features. Right subplot: layout and estimated robot trajectory.

**Figure 16:**
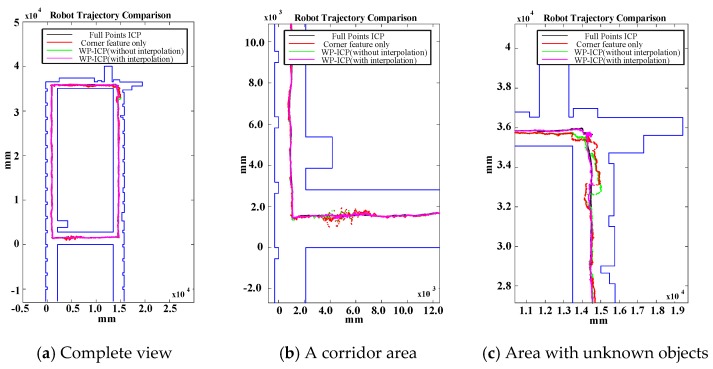
Experiments under different algorithms in the DAA-3F.

In the experiment, the traveling path of the vehicle goes counterclockwise on the 3rd floor. The transient localization behavior can refer to Figure 15, where the upper left subplot and lower left subplot shows that all the points in data set (from a LiDAR scan) and a model set (from a layout) are already being labeled by corner features and line features, respectively. Those two features are fed into the parallel ICP process and finally being fused to a single robot pose. Facing the area that is different from layout and passing through a straight corridor, the result from utilizing corner feature points based ICP algorithm leads to apparent localization deviations. Figure 16 shows a long range localization results at DAA-3F under the use of different algorithms.

Examining Figure 16 again, since WP-ICP provides both corner and line features as scan matching correspondences, theoretically the results are supposed to be better than those conducted by the corner-based ICP. However, Figure 16b indicates that there are still estimation errors when passing through the corridor. It is because of fewer line features in the area. To further improve the estimation robustness, an interpolation on the line features is further integrated into the WP-ICP. Note that the interpolation is used to increase the number of line feature points for every 10 cm. The result (i.e., the purple line) shows a good estimate when utilizing the WP-ICP algorithm with interpolation. Finally, the improvement can also be found in locations subject to static unknown objects as shown in Figure 16c, where the corresponding snapshots are shown in Figure 14a,b, respectively.

**Figure 17:**
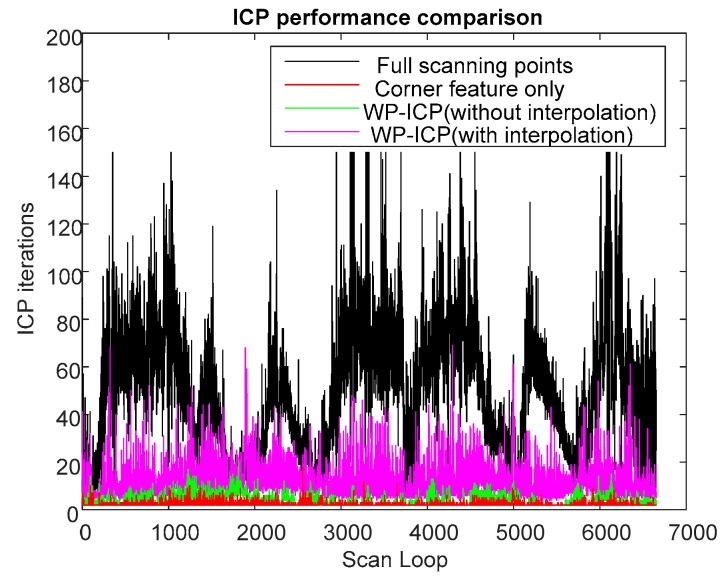
ICP performance comparison of the third experiment.

The results of WP-ICP with interpolation demonstrate the robustness against the external environment uncertainty. The details of the computation efficiency under different algorithms are depicted in Figure 17. Compared to the full point ICP, the use of corner features only can improve speed by about 50 times on average; the use of WP-ICP (with interpolation) can improve speed by 5 times on average. However, applying corner features only could sometimes lead to unstable estimates. Therefore, WP-ICP makes a compromise between computation speed and localization accuracy.

Finally, it is worth discussing the localization performance under different interpolation sizes when applying the WP-ICP. In this study, the layouts of the localization environment are given in terms of few discrete data points. Therefore, the resolution of the layouts can be further enhanced by using interpolation. For each LiDAR scan, the interpolation can be achieved by manipulating the raw data directly. The simplest way is to apply a divider on each segment.

**Figure 18:**
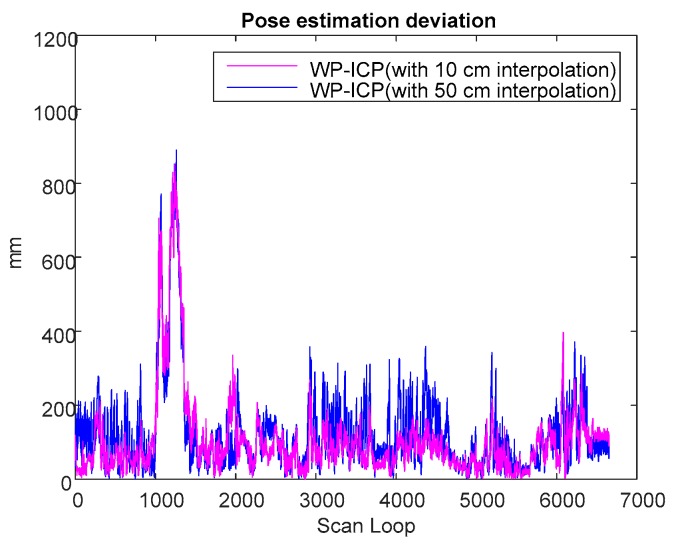
Pose estimation deviation of different interpolations.

Based on previous experiments, it is obvious that WP-ICP with interpolation has the minimum error compared with the corner-feature-based ICP and the pure WP-ICP. In the following, 10 and 50 cm interpolation resolutions are further considered for the same DAA-3F experiment. Figure 18 verifies that increasing the size of the interpolation resolution does increase the localization error. However, the number of ICP iterations can be significantly reduced. The average iterations of WP-ICP with 50 cm and 10 cm interpolations are 7.063 and 11.7577, respectively. As a result, the resolution of the interpolation can be taken as a trade-off design factor between the computation efficiency and the localization precision.

A comparison study from the viewpoint of the ICP iteration is summarized in Table 1. It is clear that the ICP iteration performances obtained by the corner-based ICP and WP-ICP are noticeably better than full-points ICP. To overcome the challenge of unknown objects during point cloud matching, WP-ICP with interpolation was further introduced. It was shown that the localization robustness can be significantly enhanced. Although the number of ICP iterations increases, the increment is still acceptable for real-time consideration.

**Figure 19:**
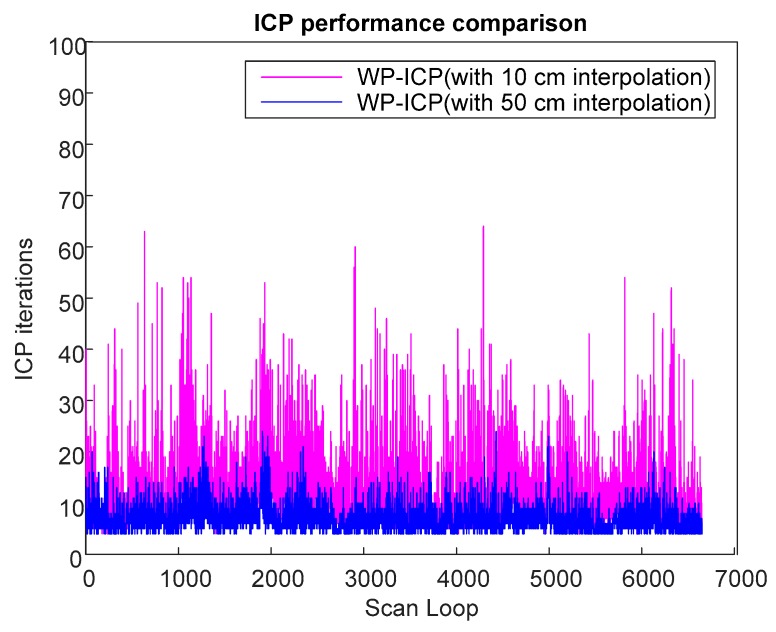
ICP performance of different interpolations.

**Figure 20:**
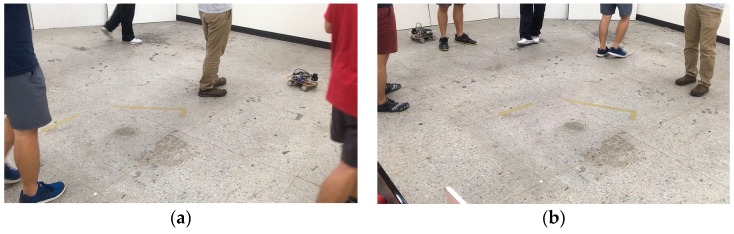
An environment with unknown moving objects. (**a**) Random snapshot 1. (**b**) Random snapshot 2.

**Figure 21:**
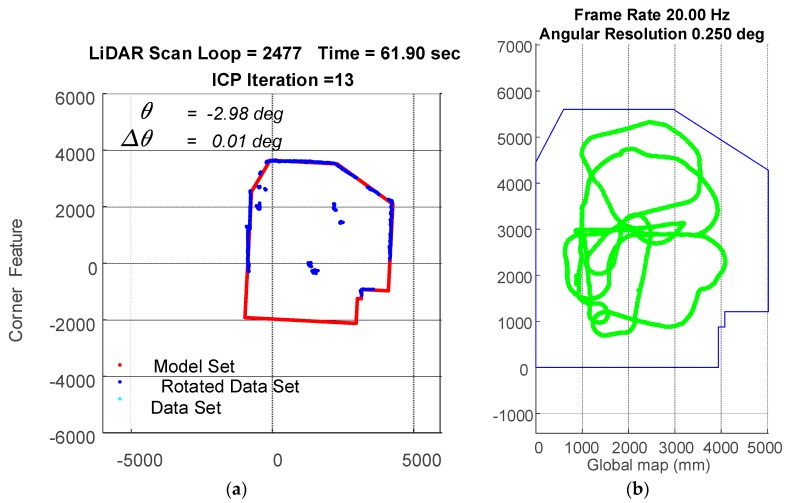
Localization result by using full-points ICP. (**a**) Point registration. (**b**) Robot trajectories.

**Figure 22:**
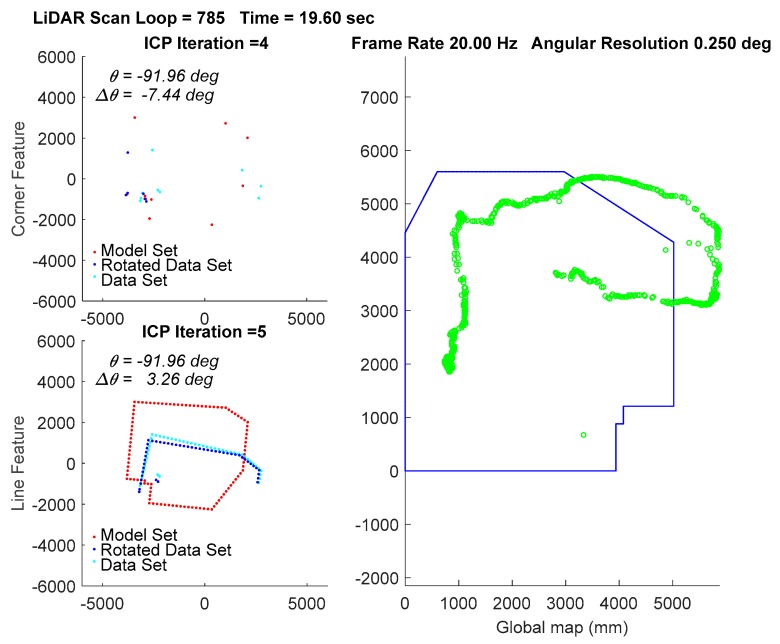
Localization result by using corner features only (where the line information was not fused for the localization).

**Figure 23:**
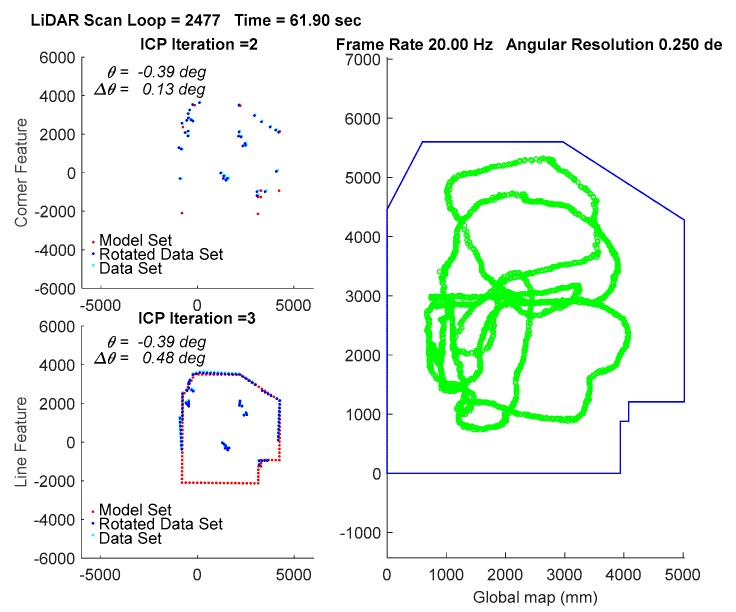
Localization result using the WP-ICP with 20 cm interpolation.

**Figure 24:**
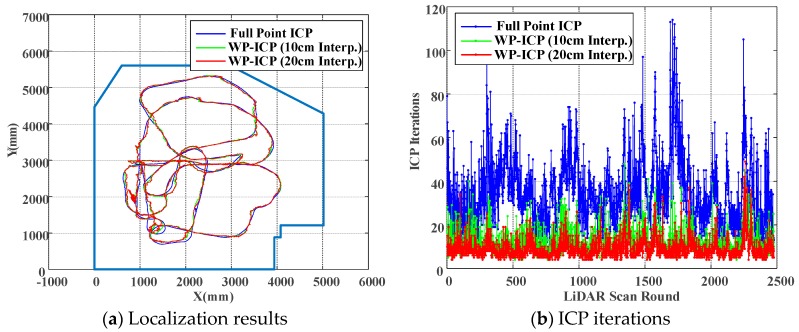
Localization results and ICP iterations.

Finally, to further demonstrate the robustness of the proposed WP-ICP, we considered another experiment in the same environment (as illustrated in Figure 19) but with five people walking around the vehicle. The practical scenes are shown in Figure 20. Firstly, to generate a ground truth for comparison, the full-points ICP is considered, which leads to good localization results, as illustrated in Figure 21. Due to many unknown moving objects that do not exist in the given layout, those obstacles result in many outliers. Under this condition, using corner features only would cause divergent localization, as demonstrated in Figure 22. On the contrary, as shown in Figure 23, applying the WP-ICP together with interpolation presents satisfactory localization results without inducing divergent behavior, even in the presence of unknown moving objects. For the WP-ICP under different interpolation resolutions, the results are depicted in Figure 24a,b, respectively. The performance details are given in Table 2. Experiments verify that the use of WP-ICP can withstand dynamic uncertainties as well as produce satisfactory localization result with fewer iterations.

## 5. Conclusions

In this work, to solve the mismatched point cloud registration problem and to enhance ICP efficiency, a parallel feature-based indoor localization algorithm is proposed. In the traditional ICP algorithm, the point cloud registration is achieved by means of NNS and there is no any other information attached to those points. Therefore, it is prone to obtain incorrect correspondences. On the contrary, we present a novel WP-ICP algorithm that provides more information on the polished point cloud. The WP-ICP consists of two ICP sources, one is corner features and the other is line features. Owing to the parallel mechanism, it attenuates mismatch probabilities from corner to line matching or from line to corner matching. As a result, the proposed algorithm results in faster convergence for pose estimation. Moreover, since the full scan points are processed to extract fewer feature points, it also enhances the ICP computation efficiency and therefore is suitable for low-cost CPUs. For environments that possess fewer feature points, the WP-ICP together with a line interpolation is further verified. Environments subject to static and dynamic unknown moving objects were also considered to verify the feasibility and robustness of the proposed method.

## Figures and Tables

**Table 1:** ICP iteration performance.

Experimental Environment	Pose Estimation Algorithm	Average ICP Iteration
**Scene-1 Exp.1**	Full-Points ICP	25.36
	Corner-Based ICP	2.06
	WP-ICP	2.06/2.04 (corner/line)
**Scene-1 Exp.2**	Full-Points ICP	30.10
	Corner-Based ICP	2.04
	WP-ICP	2.05/2.04 (corner/line)
**Scene-2**	Full-Points ICP	49.92
	Corner-Based ICP	2.49
	WP-ICP	2.61/2.85 (corner/line)
	WP-ICP with 10cm Interpolation	2.87/9.25 (corner/line)

**Table 2:** Localization results for the environment subject to unknown moving objects.

Localization Algorithm	Model Set Size (Points)	Average ICP Iterations	Total ICP Iterations	Local. Error (mm) Avg/Max
**Full-Points ICP (Point-to-Point ICP)**	1991	35	86687	taken as ground truth
**WP-ICP with 10 cm Interpolation**	213	13	30838	53.7/261.3
**WP-ICP with 20 cm Interpolation**	114	9	22981	60.5/264.6
